# Supplementary material for: Are carbon emissions trading and green financial instruments synergistic? -Comprehensive quantitative research based on content analysis
Source: PLoS One. 2024 Mar 7;19(3):e0298601. doi: 10.1371/journal.pone.0298601 (PMC10919601; doi:10.1371/journal.pone.0298601)
Supplement: S1 Data — (ZIP) [file pone.0298601.s001.zip › Data/地级市Z_F工作报告.zip!/░▓╗╒/╨√│╟╩╨/└┤╫╘╣1⁄2╓┌║┼í╛┬φ┐╦╩2╛▌═°í┐_2018.docx]

——2018年1月4日在宣城市第四届

人民代表大会第二次会议上

宣城市人民政府市长	张冬云

各位代表：

现在，我代表市人民政府，向大会报告政府工作，请予审议，并请市政协各位委员提出意见。

一、2017年工作回顾

2017年是新一届政府工作的开局之年。全市上下在市委的坚强领导下，深入学习贯彻党的十九大精神，以习近平新时代中国特色社会主义思想为指导，认真落实省委、省政府和市委决策部署，围绕“235”工作总体布局和“向苏浙对齐、在全省争先”工作总要求，统筹推进各项工作，圆满完成市四届人大一次会议确定的目标任务。全年完成地区生产总值1188.6亿元，增长8.5%；财政收入220.2亿元，增长8.8%；城镇、农村居民人均可支配收入分别达33548元和14590元，均居全省第4位，分别增长8.7%和9.1%；新增城镇就业5.8万人，登记失业率3.03%，低于省控制指标1.47个百分点。主要经济指标增幅好于预期、高于全国全省，经济发展质量和效益进一步提升，预期能够实现“保八争七”基础上的争先进位。尤其是经过多年持续推进“六年并进、六城同创”，解决了许多长期想解决而没有解决的难题，办成了许多过去想办而没有办成的大事，开创了宣城现代化“四个特色之城”建设新局面。

一年来，我们重点做了以下工作：

（一）坚持精准施策，实体经济加快发展。丰富“1+N”政策体系，开展“四送一服”双千工程、帮扶“四企一项目”活动和减税清费降本行动，有力促进了实体经济发展。“去降补”成效明显。规模以上工业总产值超过2000亿元、增加值增长9.2%，居全省第8位，主导产业产值超过1000亿元。企业支出减少4.9亿元，56户困难企业扭亏为盈。民营经济固定资产投资超过1000亿元，民营经济专项考核居全省第6位，较上年提升3个位次。房地产市场平稳健康发展，商品住宅去化周期缩短5个月。有效畅通金融血脉，发放新型政银担贷款62亿元，新增贷款185亿元，创历史新高；完成直接融资98亿元，郎溪华菱、广德广信、宁国晨光上市融资实现预期，新增“新三板”挂牌企业2家。现代服务业发展提速。服务业增加值增长9.7%。新增省级服务业集聚区2家。全域旅游示范区创建和旅游目的地建设扎实推进。全年接待国内外游客3135万人次，实现旅游总收入270亿元，增幅分别居全省第2位和第1位。完成“皖南川藏线”旅游度假区概念性总体规划编制。获批国家级风景名胜区1个，创成4A级景区2个。荣获全国旅游“厕所革命”先进市称号。建成全省首家旅游大数据中心，智慧旅游等级评定居全省首位。宣城文旅集团正式运营。成功举办第八届文房四宝文化旅游节，首次推出“文房四小宝”旅游文创产品。实现社会消费品零售总额531.5亿元，增长11.7%。新增限上商贸流通企业59家。电子商务进农村实现全覆盖，“上街去”网络科技成为商务部电子商务示范企业。民生银行宣城分行上报待批。现代农业稳步发展。实现农产品加工产值810亿元，增长9.5%。粮食总产133.2万吨。宣城现代功能农业科创园启动建设。新增省级示范合作社9个、示范家庭农场25个、示范农业产业化联合体16个。创建部（省）级畜禽养殖标准化示范场4个。新增“三品一标”产品62个。项目建设提质提效。完成固定资产投资1580.5亿元，增长11.8%，居全省第7位。列入省亿元以上重点项目完成投资540亿元，项目开工率、竣工率、投资完成率均居全省前列。古泉换流站、绩溪抽水蓄能电站节点工程建成。落地PPP项目22个，居全省第3位。

（二）坚持创新驱动，发展动能不断增强。科技创新潜力居全省前列。宛陵科创城启动建设，安徽工业大学宣城工业技术研究院、安徽工程大学宣城产业技术研究院、机械科学研究总院宣城产业研究院、中澳新能源材料与器件研发中心、科大讯飞宣城人工智能研究院等21家创新平台签约落户。与50多家知名院所对接产学研合作项目220项。宣州经开区转型更名为省级宣城高新区并正式申报国家级高新区。新建省级以上创新平台16家，宣城智谷产业园、0563创客街获批国家级众创空间。研发经费投入强度增幅居全省第1位，万人发明专利拥有量6.6件，增幅居全省第4位。获得国家专利优秀奖1项。新认定高新技术企业85家，居全省第6位；高新技术产业增加值、战略性新兴产业产值增长17.1%和25%，分别居全省第7位和第8位。招引高层次人才团队20个，6家团队获省扶持，居全省第3位。成功举办宣城宛陵科创城首届创新创业大赛。宁国成为全国中小企业知识产权战略推进工程试点城市。先进制造业加快发展。中国制造2025宣城行动方案全面实施。开展智能制造示范工程，5户企业获国家智能制造、绿色制造专项，11户企业智能工厂和数字化车间获省认定，新增省级以上两化融合贯标试点企业13户、国家级两化融合贯标达标企业4户，均居全省前列；推广应用工业机器人908台。旌德建成全省首个健康智造中心。持续开展“千企升级”计划，工业技改投资增长26.2%，居全省第5位。司尔特肥业跻身国家级制造业和“互联网+”融合发展示范企业，博瑞特公司成为国家级首批“服务制造示范”平台。质量强市成效显著。新增安徽名牌16个、安徽工业精品7个。广德、泾县被评为省质量强县。新增注册商标1712件，有效注册商标突破一万件。新注册地理标志证明商标4个，总数居全省第3位。2个产品获得国家生态原产地产品保护。绩溪“金山时雨”成为国家级农产品地理标志示范样板。“旌德灵芝”获评国家农产品地理标志。

（三）坚持统筹协调，城乡发展步伐加快。城市品牌更加彰显。众志成城、全民参与，首创首成第五届全国文明城市，荣获全省唯一的首批国家生态文明建设示范市和全省第三个国家卫生城市，一年内同时获得三个国家级城市品牌是安徽唯一。市本级产业基础不断夯实。宣城经开区主要经济指标增幅保持全市前列，以锂电池为主导的新能源新材料产业加快集聚，产业链逐步完善，落户企业41家，总投资90亿元的汉能移动能源一期、华威铜箔、益佳通等重点项目开工建设或投产。宣城现代服务业产业园区发展提速，农批市场交易额突破百亿，引进电商等新经济项目18个，建材（钢材）物流交易中心、冷链物流等项目加快建设。敬亭山旅游度假区5A景区创建工作扎实开展，石涛纪念馆建成开放。中心城市建设开启新篇。《宣城市城市总体规划（2016—2030年）》获省政府批复，中心城区“双百”规模和芜马宣城市组群发展正式确立。在全省率先完成城市空间特色规划，编制完成《宣城市空间规划（2016-2030年）》《芜马宣城市组群宣城部分城镇体系规划》。续建和新建城建项目213个，完成投资195.6亿元。水阳江大道闭合段东段等10条市政道路建成通车，城市备用水源工程建成，历史文化街区等8个项目竣工交付，阳德路、创业路等重点项目开工建设，宛陵湖东湖、全民健身中心等项目进展加快。以大拆迁推动城市大建设，全市征迁拆违180万平方米，其中市区开展25次“清零”行动，完成征迁拆违近100万平方米，是上年的1.8倍。北门棚户区改造、中国茶府等历史遗留问题依法有效化解。县域经济发展得到提升。宁国、宣州、广德分别跻身2017年全国中小城市综合实力、投资潜力百强县（市、区）。宁国经开区成为工信部首批绿色园区。广德经开区列入国家级经开区培育名单。特色小镇建设加快，宁国港口镇跻身第二批全国特色小镇；入选省级特色小镇3个，居全省第1位；入选省级特色小镇（试验）1个。4个乡镇跻身2017年全国综合实力千强镇。综合交通枢纽已现雏形。县县通高铁、县县通高速迈出实质性步伐。宣绩高铁项目进行了可研审查，芜宣机场即将开工，巷口桥铁路二级物流基地获批。商合杭高铁、皖赣铁路扩能改造、杭黄高铁、芜湖至广德铁路电气化改造等工程进展顺利，杭临绩高铁、宣镇高铁前期工作快速推进。交通建设完成投资63亿元，国省干线好路率连续三年居全省首位。宣狸高速建成通车，广宁高速进展顺利，芜黄高速宣城段在旌德率先开工建设，宣泾高速完成工可行业审查。宣城客运总站、宣州综合码头工程一期完成主体工程。水阳江航道整治、绩谭旅游快速通道工程有序推进。建成S214定十路和S215绩溪至歙县段一级公路。完成农村道路畅通工程1558公里。水利、电力等基础设施建设加快。双桥联圩、城东联圩堤防除险加固工程基本完工，敬亭圩排涝泵站和水系整治工程开工建设，城区防洪标准由10年一遇提高到50年一遇。青弋江治理、扬溪源水库等工程加快推进，水毁修复和薄弱环节建设治理任务超额完成。郎溪第一联合圩除险加固工程全面完工。重点水利工程投资完成率全省第二。新建、续建输变电工程12项、投产9项，完成农网改造升级“两年攻坚战”任务，新建和改造高低压线路2840多公里，惠及低压用户4.8万户。安徽电信首批建设的宣城省级云计算中心投入使用。

（四）坚持生态优先，人居环境持续改善。生态文明建设成效显著。成为国家低碳试点城市和全国绿色发展优秀城市。旌德被命名为全国“绿水青山就是金山银山”实践创新基地，绩溪成为首批国家生态文明建设示范县，被评为“中国天然氧吧”。省政府办公厅专门通报表彰我市和绩溪、旌德生态文明建设工作，并予以1600万元奖补。河长制实现市县乡村四级全覆盖。绩溪在全省率先推行民间河长制、河警制。全国绿化模范城市创建扎实推进，在全省率先试点林长制，林业“增绿增效”行动启动实施。广德创成国家园林城市。环境质量继续保持良好。深入实施“蓝天行动计划”，加强秸秆禁烧和综合利用，空气质量居全省第2位。禁养区内472个畜禽养殖场全部搬迁或关闭。地表水水质优良比例达80%，11个县级以上集中式饮用水水源地水质全部达标。率先启动市县土壤污染治理与修复规划编制工作。完成市县乡土地利用总体规划调整完善工作。报批建设用地2.8万亩，保障了项目建设。中央和省环保督察、国家土地例行督察反馈问题年度整改任务如期完成。美丽乡村建设得到加强。乡镇政府驻地建成区整治、中心村建设、自然村环境整治和“三线四边”环境治理扎实开展，美丽乡镇和省级中心村整体进度达96%。全面实施农村环境“三大革命”，80个乡镇、720个村集中开展陈年垃圾清理，建设乡镇污水处理设施17个，改造非卫生厕所2.6万户。宁国列入首批全国农村生活垃圾分类和资源化利用示范县，宁国狮桥村、绩溪仁里村成为全国改善农村人居环境示范村。

（五）坚持改革开放，发展动力进一步增强。年度改革任务全面完成。农村“三变”改革、公立医院管理和费用控制“双百”综合考评等改革举措在全国有影响。加大“放管服”改革力度，公布市级公共服务清单，行政审批中介服务清理精简幅度位于全省前列，“双随机一公开”监管实现全覆盖。商事制度改革持续深化，新登记市场主体3.1万户，增长6.8%。城市大建设、项目建设和招商引资工作指挥调度机制高效运行。市属国有平台公司改革转型为实体企业，宣城现代服务业产业园区实行市场化运作、公司化运营。完成城市总规编制审批改革创新省部试点，成为全国城市设计试点城市。开展信用试点工作，建成市信用信息共享服务平台。创新工商联服务非公经济工作体制。在全省率先实现“一窗一人”联合办税全覆盖，纳税人满意度全省第一，获评全国百佳国税地税合作市级示范区。农村土地“三权分置”改革扎实推进，200个村完成农村集体产权制度改革，97个村实施农村“三变”改革，农村产权交易平台上线运营。旌德全国农村集体产权制度改革试点、宣州全国农房抵押贷款试点和农村综合性改革试点进展加快。小型水利产权制度改革、国有林场改革全面完成。荣获全国集体林权改革先进集体称号。推进城市执法体制改革，市城市管理综合执法局正式挂牌。招商合作力度加大。深化与沪苏浙重点城市对接合作，宣城新塘羽绒产业园建设提速，苏皖合作示范区、沪宣“飞地”合作等项目加快推进。强化产业招商，新引进省外投资亿元以上项目270个，居全省首位；亿元以上项目到位省外资金883.3亿元，增长14.7%，主导产业契合度达59.3%；实际利用外资9.2亿美元，增长7.5%。宁国紫燕食品、国家机动车监督检验中心广德基地、郎溪国肽生物等重大项目成功引进，一批产业类重大项目在手洽谈。外向型经济水平稳步提升。进出口总额15.3亿美元，居全省第7位。口岸建设加快，宣城无水港业务进一步发展，皖东南保税物流中心（B型）、中鼎公用型保税仓库即将封关运行。新增进出口实绩企业102家、境外投资企业4家，新设外资企业15家。外贸结构进一步优化，跨境电子商务进一步发展。

（六）坚持共建共享，群众获得感显著提升。脱贫攻坚强力推进。实施脱贫攻坚“八项行动”，开展“重精准、补短板、促攻坚”和大排查专项行动，实施到村产业项目553个、到户6.6万个，贫困人口脱贫年度任务如期完成，剩余97个贫困村全部出列；提前完成“十三五”期间易地扶贫搬迁任务，54户104人全部入住新居。民生保障不断加强。完成省定33项民生工程和市政府10件民生实事。超额完成社会保险参保任务，机关事业单位养老保险改革稳妥实施。农村五保供养标准进一步提高，城乡居民低保制度持续完善，城乡医疗救助、社会救助能力得到增强。棚户区改造新开工14548套，基本建成15374套。综合整治改造老旧小区41个，改造农村危房6495户。解决16069名农村居民安全饮水问题。健全养老服务体系，开展养老院服务质量建设专项行动。社会事业短板加快补齐。义务教育发展基本均衡县实现全覆盖。十五年一贯制的北京师范大学宣城学校正式落户。新建改建13所幼儿园，市第九小学建成使用，市二中改扩建工程完工。合工大宣城校区基础设施建设扎实推进。安徽工业技师学院筹建工作有序开展。民办教育、中等职业教育加快发展。教师“无校籍”管理稳步推进。医疗服务体系进一步完善，市人民医院创成三甲医院、医技大楼建成，宣城迪安医学诊断中心建成执业，市中心血站血液检测楼开工建设，县域医共体试点实现全覆盖。皖南医药卫生学校启动建设，与中国科技大学第一附属医院即省立医院合作建设皖南分院（即市妇幼保健院）。成为全国首批社会办中医试点市。宁国跻身国家卫生城市。全面加强人口计生工作，人口自然增长率在5‰以内。市规划馆、博物馆、宣城书法馆正式开馆。建成乡镇（社区）综合文化服务中心24个、农民文化乐园46个，数字化农家书屋建设在全省率先启动。成功举办市第二届全民健身运动会，圆满完成亚洲山地自行车锦标赛等国际国内重大体育赛事承办工作。宣州区获评全国群众体育先进单位。第三次全国农业普查工作顺利完成。妇女儿童、老龄、红十字等事业进一步发展，外事侨务、对台、机关事务管理、档案、地方志、科普等工作取得新成绩。社会治理能力稳步提升。成为全省综治工作先进市、社会治安防控体系建设先进市，群众安全感和政法队伍执法工作满意度分别居全省第1位和第3位。十九大维稳安保工作获省表彰。成功处置广德“10.17”液化天然气罐车泄漏重大事故。宁国荣获全国社会治安综合治理“长安杯”。绩溪跻身全国法治县创建活动先进单位。市信访局荣获全国信访系统先进集体。深入推进“七五”普法，实施法治家庭“百千万”工程。民族宗教工作平稳有序。深化“铸安”行动，安全生产形势持续平稳。广德成为全国安全生产先进单位。食品药品安全城市创建积极开展。应急指挥体系建设得到加强，防灾减灾救灾能力稳步增强，汛期灾害损失为历年最低。新一代多普勒天气雷达投入试运行。新一轮省级双拥模范城创建通过验收，“宣城舰”正式入列，优抚安置工作取得新进展，国防动员、民兵预备役、人民防空工作进一步加强。

（七）坚持不忘初心，加强政府自身建设。始终强化政治定力。牢固树立“四个意识”，深入学习贯彻党的十九大精神，扎实推进“两学一做”学习教育常态化制度化，认真开展“讲政治、重规矩、作表率”专题警示教育，把讲政治落实到政府工作各方面。政府法治工作进一步加强。完善市政府工作规则，严格执行重大行政决策程序规定，强化公众参与、合法性审查工作，促进决策规范透明。完成地方立法项目的起草和制定工作。自觉接受市人大及其常委会的法律监督、工作监督和市政协民主监督。办理市人代会议案和建议103件、市政协委员提案270件。坚持以法治思维和法治方式深化改革、推进发展、化解矛盾、维护稳定，行政机关负责人出庭应诉率居全省前列。强化工作推进落实。坚持项目建设“四督四保”制度，实行重大项目每两月观摩调度、每季度集中开工竣工投产工作机制，建立完善经济运行调度通报考核、政务督查考核等制度，设立“无会周”，推进各项工作落实和外出招商。群众对政府总体工作满意度居全省第2位。加强廉政勤政建设。强化政府效能建设，大力推进“互联网+政务服务”，深化政务公开，努力打造“四最”营商环境。网上政务服务平台上线运行，率先完成“12345”政府服务热线整合。完善审计监督全覆盖机制，加强财政资金监管，“三公”经费等一般性支出进一步压缩。积极做好巡视巡察反馈问题整改工作，支持纪检监察机关开展工作，持续正风肃纪。整饬“四风”常抓不懈，严厉查处违反中央八项规定精神问题；开展扶贫领域专项整治，坚决整治侵害群众利益的不正之风。

各位代表！回首过去的一年，我们深刻感受到，加快宣城的发展，必须坚持党对经济工作的领导，以习近平新时代中国特色社会主义思想为根本遵循，坚定贯彻落实党中央和省、市委的各项决策部署；必须坚持以人民为中心的发展思想，把握社会主要矛盾变化新特点，牢牢抓住发展第一要务，持续加强和保障民生，努力满足人民日益增长的美好生活需要；必须坚持新发展理念，实施创新驱动发展战略，全面深化改革，持续扩大开放，坚持绿水青山与金山银山有机统一，推动实现高质量发展。

各位代表！过去一年成绩的取得实属不易。这是省委、省政府和市委坚强领导的结果，是市人大、市政协监督支持的结果，是全市人民加油实干的结果。在此，我代表市人民政府，向全市人民，向全体人大代表、政协委员，向各民主党派、工商联、各人民团体和各界人士，向驻宣解放军指战员、武警官兵和政法公安干警，向关心、支持宣城发展的中央和省驻宣单位，向在我市投资创业的广大企业家、建设者，表示衷心的感谢和崇高的敬意！

在肯定成绩的同时，我们清醒认识到，全市发展还存在不平衡不充分问题。主要表现在：新发展理念落实还有差距，发展质量和效益还不够高，内生增长动力仍显不足；城乡发展尚不平衡，中心城市建设任务艰巨，县域经济发展需要增量提质；经济外向水平有待提升，对外开放通道、平台和大通关建设亟待加强；生态建设力度需要进一步加大，环境治理任务仍然较重；社会事业和民生领域还有不少短板，脱贫攻坚成果还需要巩固提升；制约发展的深层次体制机制弊端尚未根本破除，少数公职人员的理念、能力、作风和精神状态与新时代发展要求还不能完全适应，履职能力和本领需要提升。对此，我们将认真整改，绝不辜负人民群众的期待。

二、2018年工作安排

2018年是贯彻党的十九大精神的开局之年，是改革开放40周年，是决胜全面建成小康社会、实施“十三五”规划承上启下的关键一年。做好今年改革发展稳定工作，意义重大、影响深远。要全面贯彻党的十九大和中央经济工作会议精神，以习近平新时代中国特色社会主义思想为指导，深入贯彻习近平总书记视察安徽重要讲话精神，落实省委十届六次全会、省委经济工作会议和市委四届五次全会、市委经济工作会议决策部署,坚持稳中求进工作总基调，坚持新发展理念，紧扣社会主要矛盾变化，按照高质量发展的要求，坚持以供给侧结构性改革为主线，以落实“五大发展行动计划”、建设现代化“四个特色之城”为总抓手，围绕“235”工作总体布局和“向苏浙对齐、在全省争先”工作总要求，大力实施“六大战略”和6个“1号工程”，推动质量变革、效率变革、动力变革，在打好“三大攻坚战”方面取得扎实进展，统筹做好稳增长、促改革、调结构、惠民生、防风险各项工作，促进经济社会持续健康发展，为决胜全面建成小康社会奠定坚实基础。

立足宣城实际，我们要在保持一定发展速度基础上，聚焦推动高质量发展这一根本要求。建议全市经济社会发展的主要预期目标是：地区生产总值增长8.3%左右，服务业增加值增长10%左右，财政收入与经济增长同步，规模以上工业增加值增长8.5%以上，固定资产投资增长10.5%左右，社会消费品零售总额增长10.5%左右，进出口总额增幅高于全省，城乡居民人均可支配收入增长8.5%左右，社会调查失业率控制在4.8%以下，完成省定节能减排任务。在实际执行中，力争完成得更好一些。

围绕上述目标，我们将突出抓重点、补短板、强弱项，拿出硬措施，打赢攻坚战，着力做好以下九项重点工作：

（一）坚决打好决胜全面建成小康社会三大攻坚战。研究制定未来三年攻坚规划和年度战役计划，排出时间表、路线图，明确责任主体，集中优势兵力，一仗接着一仗打，确保取得突破性进展。

坚决打好防范化解重大风险攻坚战。围绕财政金融风险总体可控，开展“四清四实”专项整治，清理市县政府隐性债务，妥善处置隐性债务存量，严控隐性债务增量，稳妥处置政府债务风险。常态化加强全口径政府举债管理，加快政府平台公司市场化转型，强化对融资平台、产业投资基金、PPP项目、政府购买服务的动态监管，落实政府举债终身问责制和债务问题倒查机制，规范政府举债行为，强化财政预算硬约束，从机制上源头上约束违规举债。健全金融风险监测预警和联合处置机制，开展非法违规金融活动打击行动、互联网金融专项整治行动、地方金融机构不良资产压降行动，积极化解不良贷款陈年旧账，多管齐下、多措并举、标本兼治，坚决守住不发生系统性金融风险的底线。

坚决打好精准脱贫攻坚战。扎实推进全面小康“1号工程”，坚持精准扶贫、精准脱贫，瞄准特定贫困群众精准帮扶，聚焦贫困难点村集中发力，加强非贫困村贫困人口脱贫工作，确保现行标准下的脱贫质量，如期完成年度减贫任务。坚持扶贫与扶志、扶智相结合，引导贫困群众树立主体意识，强化产业就业扶贫，深化产业扶贫利益联结机制，加强基层基本公共服务功能建设。完善脱贫攻坚责任体系，加强扶贫工作队伍建设，强化督查考核，进一步夯实基层基础。巩固脱贫成效，确保脱真贫、真脱贫，不断夺取脱贫攻坚新胜利。

坚决打好污染防治攻坚战。坚持全民共治、源头防治，持续推进“洁气、洁水、洁土”行动。强化工业废气污染、各类扬尘污染、非煤矿山和城区散煤、烟花爆竹燃放、餐饮油烟、机动车污染等专项整治，建立污染源在线监控体系，加强秸秆禁烧和综合利用，坚决打赢“蓝天保卫战”。完善并规范运营工业集聚区配套污水集中处理设施，推进城市污水集中处理设施提标改造。强化畜禽养殖区污染治理，完善综合利用和污染处理设施。实行最严格的水资源管理制度，巩固完善河长制，落实“一河一策”。开展集中式饮用水水源规范化和应急保障体系建设，强化入河排污口整治。完成耕地土壤环境质量类别划定，开展土壤环境状况调查与风险评估，严控工矿、农业污染源，防治新增土壤污染。推进城市生活垃圾强制分类试点，建成市生活垃圾焚烧发电厂二期。加快建立以排污许可制度为核心的固定源环境管理制度。全面完成中央环保督察反馈问题整改任务。开展第二次污染源普查。深入开展环保“绿剑行动”，坚决查处破坏生态环境行为。

（二）聚焦提高供给体系质量，加快建设创新型现代产业体系。坚持质量第一、效益优先，依靠创新着力培育发展新动能，加快产业转型升级。

大力推进科技创新。实施科技创新“1号工程”，加快宛陵科创城一期工程建设，建成7.6万平方米，启动人才公寓建设，力争已引进科研院所年内入驻。加快创新平台建设，新增省级以上创新平台10家以上。统筹推进“三重一创”建设，加快战略性新兴产业集聚基地建设和高新技术企业发展，新认定高新技术企业35家，战略性新兴产业产值、高新技术产业增加值分别增长12%和15%左右。加大科技创新投入，力争研发经费投入强度达1.6%。万人发明专利拥有量达7.5件以上。持续创建省级创新型试点城市。加强创新人才队伍和新型智库建设，引进高层次科创团队10个以上，争取省支持团队3个。继续实施“宣城籍优秀人才回归工程”。大力开展科学普及工作。举办第二届中国宛陵科创城创新创业大赛、中国宣城大学生创新创业大赛、宣城青少年科技创新大赛。

加快制造强市建设。推进中国制造2025宣城行动，争创中国制造2025国家级示范区。实施“百亿企业、千亿产业”工程，加快培育汽车及装备制造、新能源新材料、食品医药和电子信息“3个千亿产业”。以智能装备、电子信息、汽车和新能源汽车等为重点，做大做强高端制造业。积极推进产业发展“1号工程”，发展壮大新能源新材料产业，加快汉能移动能源、华威铜箔、赛德丽新能源等重点项目建设，推动已落户企业投产达效，加速形成产业集群和上下游配套。加强与中船重工集团714所战略合作，促进军民深度融合发展。

积极发展智慧经济。加快宣城智谷产业园建设，推进科大讯飞人工智能宣城产业基地、“宣城e谷”和智慧教育示范区建设，努力打造全省重要的智慧经济发展基地。推动互联网、大数据、人工智能和实体经济深度融合，加快智慧城市建设，积极培育发展智慧交通、智慧物流、智慧医疗等产业。

提升质量强市建设水平。开展质量提升行动，鼓励和引导企业争创质量品牌，新增商标注册申请2200件，力争主导或参与制定国家标准、行业标准、省级地方标准10项以上。新增安徽工业精品5个。开展市政府质量奖评选。加强国家宣纸及文房用品质量监督检验中心建设。支持宁国创建全国质量强市示范城市。推进泾县国家级电机检测检验中心建设。

（三）着力壮大实体经济，切实筑牢发展根基。把发展经济的着力点放在实体经济上，大力推进供给侧结构性改革，增强实体经济发展活力。

扶育培强实体企业。持续开展“四送一服”双千工程，落实兑现“1+N”促进政策。坚决处置“僵尸企业”，鼓励企业兼并重组，淘汰落后产能，推动化解过剩产能。继续开展减税清费降本行动，加大对乱收费的查处和整治力度，降低企业制度性交易成本和用能、物流成本。实施行业龙头骨干企业、“科技小巨人”企业培育工程，力争新增省“专精特新”企业25户。大力促进小微企业成长，新增工业企业“小升规”30家、限上商贸流通企业20家。

加大项目建设力度。深入开展“项目提质提效年”活动，严格落实“四督四保”“三查三单”制度，继续开展重大项目集中开工竣工投产活动。加大科技创新、产业发展、基础设施、公共服务、生态环境等领域补短板力度，力争实施亿元以上重点项目600个，完成投资600亿元以上；其中新开工200个、竣工150个。确保古泉换流站竣工运行、绩溪抽水蓄能电站下水库蓄水。

千方百计保障要素。加大金融服务实体经济力度，力争新增贷款130亿元，提高对制造业、小微企业、“三农”等重点领域信贷投放比重。鼓励金融产品创新，推广林权、仓单、应收账款、存货等抵质押信贷产品。继续推进企业上市“翱翔计划”，新增上市企业1家、“新三板”挂牌企业3家，实现直接融资80亿元以上。规范运作政府性产业投资引导基金，培育发展新兴产业。加强存量用地盘活和批而未供土地清理工作，推进国土资源节约集约模范市创建。扎实开展新一轮土地利用总体规划修编国家试点。开展第三次全国土地调查工作。加强技能型人才培养，培育“宣城工匠”，推进“技工大市”建设。搭建企业用工供需平台，出台奖补政策，优化招工服务，强化技能培训，缓解企业用工困难。

（四）深化重点领域改革，持续释放发展活力。坚持敢闯敢试、攻坚克难，进一步深化体制机制改革，坚定不移向各领域各环节各层次纵深推进。

促进民营经济发展。深化“放管服”改革，优化政府权责清单和涉企收费清单，完善公共服务清单和中介服务清单，启动实施市场准入负面清单制度，强化“双随机一公开”监管。深化商事制度改革，探索便利化登记服务，力争新登记民营企业3600户、个体工商户1.4万户以上。扩大重点领域社会投资，推进政府与社会资本合作，促进民间投资稳定增长。弘扬劳模精神和企业家精神，开展市第五届优秀中国特色社会主义事业建设者评选表彰活动；依法平等保护各类产权，构建亲清新型政商关系，让各类市场主体创业愉快、发展愉快、生活愉快。

深化社会保障制度改革。加大探索创新力度，稳妥推进住房制度改革，加快建立多主体供应、多渠道保障、租购并举的住房制度。支持群众改善性住房需求，加快推进非住宅类商品房去库存，促进房地产业平稳健康发展。推进机关事业单位养老保险制度改革，整合城乡居民基本医疗保险制度。深化医药卫生体制综合改革，建立公立医院灵活用人制度，全面推进公立医院和乡镇卫生院编制周转池制度改革。加快医联体、医共体发展。深化医保付费方式改革，推进分级诊疗和家庭医生签约服务，提高服务水平。

统筹推进其他各项改革创新。深化预算管理改革，推行财政支出绩效管理机制。理顺市与宣州区、宣城经开区、宣城现代服务业产业园区管理机制和财税体制。探索开展市县乡职能相近部门整合。推进公务员分类管理改革。开展生产经营类事业单位改革。稳妥推进文化市场综合执法改革和供销社综合改革。深化城市管理执法改革、统计管理体制改革和旅游综合管理体制改革。推进宁国全国殡葬综合改革试点。改革社会组织管理制度。深入推进社会信用体系建设，加强行业信用建设，实施守信联合激励和失信联合惩戒。加快市属国有公司转型发展。

（五）实施乡村振兴战略，建设新时代美丽乡村。坚持农业农村优先发展，实施乡村振兴“1号工程”，深化农村“三变”改革，加快建设“产业兴旺、生态宜居、乡风文明、治理有效、生活富裕”的农业农村现代化。

深化农村各项改革。推进农村集体产权制度改革，有序开展村集体经营性资产股份合作制改革，落实农民财产权，增强集体经济发展能力。巩固农村土地“三权分置”改革成果，加快房地一体的农村宅基地和集体建设用地确权登记发证，积极盘活空闲农房及宅基地。总结宣州农民住房财产权抵押贷款试点经验。深化集体林权制度改革。探索特色小镇发展的机制模式，加大市级特色小镇培育力度，引导特色小镇健康发展。继续抓好旌德白地镇、宁国港口镇等国家和省级特色小镇建设。推进经济发达镇行政管理体制改革，力争更多跻身中国特色小城镇。

加快发展现代农业。把增加绿色优质农产品供给放在突出位置，加快实施农业产业化“五大行动”方案，大力发展农产品加工业。持续稳定粮食生产。发展多种形式适度规模经营，培育壮大新型农业经营主体和服务主体，加快构建现代农业产业、生产和经营体系，促进小农户和现代农业发展有机衔接。争创省级示范合作社6个、示范家庭农场15个、粮食产后服务中心4个。推进农旅融合，大力发展乡村旅游，创建省级以上休闲农业示范点1个，鼓励发展田园综合体。推进农业科技创新，加快宣城现代功能农业科创园等园区建设。实施茶产业提升和竹产业、木本油料和林下中药材为主导的特色林业产业工程，加快畜牧业转型升级。推进农产品标准化生产，实施“生态宣城·绿色产品”品牌工程，新增“三品一标”产品50个。培养造就一支懂农业、爱农村、爱农民的“三农”工作队伍。

改善生产生活条件。加强农田水利建设，强化灾后水利薄弱环节建设性治理，开工建设水阳江中游综合治理工程，力争港口湾水库灌区、牛岭水库开工，加快推进凤凰山水库、汤村水库等重点项目前期工作。推进国省道和“四好”农村公路建设，新增一级公路36 公里，完成国省道路面改善和生命安全防护工程 200公里、农村道路畅通工程760 公里。加强城网农网提升改造，续建、新建220千伏江塘变、110千伏榔桥变等12个项目。稳步推进“放心粮油”和“主食厨房”工程，保障粮油消费安全和品质提升。抓好乡镇政府驻地建成区整治、中心村建设和自然村环境整治“三大任务”，持续推进农村环境“三大革命”，建成22个乡镇污水处理设施，实现覆盖率超过50%，改造3.45万户非卫生厕所。健全农村环境整治长效机制。开展全国农村生活污水治理示范县争创工作。

（六）推动区域协调发展，提升全市发展整体效能。完善区域发展战略，积极推进行政区划调整，持续开展“六年并进、六城同创”，加强“一区两廊”建设，推动市本级和县域协调联动发展。

提升中心城区发展水平。强化市区一体化发展，大力推进“多规合一”、产城融合，增强产业支撑。加快国家级宣城经开区发展，新开工项目30个、竣工投产26个，建设现代新型材料化工产业园、生物医药大健康产业园、中德产业园。力争宣城高新区升格为国家级高新区。围绕培育5个“百亿产业”，加快宣城现代服务业产业园区发展，建成电子商务与快递业融合发展集中区，力争新塘羽绒产业园新开工项目6家、投产5家，建成冷链物流大市场一期、建材（钢材）物流交易中心和农批市场二期。扎实推进敬亭山旅游度假区5A景区创建，实施山水诗舍度假民宿等项目建设。

加快“两廊”建设发展。县域经济是全市发展的支撑，必须始终紧抓不放、再铸辉煌。以“两廊”建设为抓手，推动县域经济转型升级，着力打造比肩江浙的经济强县、旅游文化名县、生态示范强县。编制发展规划，制定建设方案，加快宣郎广宁智创走廊建设。推进广德经开区升格国家级、郎溪经开区争创省级高新区。支持宣州、宁国巩固全国“百强县（区）”发展成果和国家级宁国经开区、省级宣城高新区创新转型发展，支持郎溪、广德冲刺“百强县”。加快广德撤县设市步伐。充分发挥生态、文化和旅游资源优势，加快打造泾旌绩文化旅游生态健康产业走廊。支持泾县创建国家生态文明建设示范县，加快泾县电机泵阀、宣纸书画纸、文化旅游产业发展。支持绩溪发展食品加工、文化旅游产业和电商经济，加快打造中国最佳休闲养生目的地。支持旌德“绿水青山就是金山银山”实践创新基地建设，发展壮大大健康产业。

推进中心城区建设。开展“城市建设攻坚年”活动，实施城市品质提升“1号工程”，加强城市环境综合整治。扎实开展全国城市设计试点工作，完成西部新城、火车站站前新区、城市南部文教创意园区等城市规划设计。按照“上急需、保基本、补短板、厚弱项、提品质、有保障、能平衡”原则，实施以“五校三院三中心”为重点的200个城市建设项目，完成投资150亿元。实施畅通工程,全力推进青弋江大道、陵阳路二期等71个城市道路项目，确保城区“一环”“二环”顺利闭合，打通主城区东西向骨干通道，持续推进城区道路微循环三年行动计划。实施安居工程，有序推进城区10大板块规划建设，加快北门旧城改造、宛溪河综合整治二期和安置房建设，保障存量被拆迁户尽早回迁入住。实施活水工程，推进宛陵湖东湖、解放河等5个活水项目，一体化推进城区水环境综合整治。实施美丽工程，加快推进中央公园、体育中心等项目建设，加强城市园林绿化、市政公用设施建设与管理。继续实施防洪保安工程，全面完成城市防洪除险加固工程，进一步巩固提升防洪标准。加强土地房屋征收，确保市区完成房屋征收4000户、60万平方米以上，保障重点项目建设。推进“两治三改”专项行动，确保新增违建“零增长”、存量违建基本拆除、违法用地行为有效遏制。强化城市精细化治理，开展污水管网、黑臭水体、建筑装潢垃圾、废品收购站点等专项整治，推动数字城管向智慧城管升级。

打造综合交通枢纽。继续开展“交通建设提升年”活动，完成投资52亿元。开通运营杭黄高速铁路，加快芜宣机场和商合杭高铁、皖赣扩能改造芜宣段、芜湖至广德铁路电气化改造工程建设步伐，开工建设巷口桥铁路物流基地，力争宣绩高铁开工。推进宣镇高铁、杭临绩高铁、宣泾宿铁路前期工作。建成广宁高速，加快芜黄高速、绩谭旅游快速通道建设，开工建设宣泾高速。积极打通省际“断头路”。推进申嘉湖高速西延宁国段项目前期工作。加快水阳江航道整治和宣州综合码头建设。

（七）深入践行“两山”理念，建设绿色发展创新实验区。充分发挥生态优势，以建设安徽（宣城）绿色发展创新实验区为抓手，打造生态文明建设宣城样板。

加大生态建设保护力度。落实主体功能区规划，加强生态红线管控。启动实施国家生态文明先行示范区建设中期评估。扎实开展林业“增绿增效”行动，全面推行林长制，力争创成全国绿化模范城市。参与长江经济带战略环评，推进工业园区环境影响跟踪评级。推进水土流失综合治理，严厉打击河道非法采砂。严格保护耕地，完成补充耕地和高标准农田建设任务。完善生态文明建设考核奖励机制，试行南漪湖流域横向生态补偿制度。

加快传统产业绿色化改造。推进两化深度融合，大力发展绿色制造产业、绿色产品，建设绿色工厂、绿色园区，打造长三角绿色制造基地。以农副产品深加工、建材原材料和化工等优势传统产业为重点，继续推进“千企升级”和“双百项目”计划，确保技改投资增长10%以上。

强化绿色、循环、低碳发展。推广应用绿色技术、绿色生产，积极发展节能环保、清洁生产、清洁能源产业，推广低碳技术和产品。强化资源全面节约和循环利用，加强能源消费“双控”，扎实开展国家低碳城市试点。加强与省地矿局的战略合作，推进311地质队搬迁至宣城进程。配合开展多金属矿、页岩气（油）等矿产资源深度勘查开发工作。争创国家节水型城市。倡导绿色消费、绿色出行，创建节约型机关。

提升文化旅游产业。实施文化产业创新驱动工程，推进文房四宝特色产业园区建设，创成省级文化产业园区（基地）。扎实开展“文化旅游发展提升年”活动，力争接待游客数、旅游总收入分别增长17%和19%以上。围绕打造知名旅游目的地，加快皖南国际文化旅游示范区建设，创成国家全域旅游示范区。推进全域旅游“1号工程”，实施“皖南川藏线三年行动计划”，加强云岭红色旅游景区等5A创建。强化资源整合，促进市、县文旅集团发展。完善旅游基础设施，加快五星级宾馆建设，推进“旅游厕所革命新三年行动计划”，实施景区“最后一公里”通达工程、旅游集散中心建设工程、智慧旅游提升工程。

促进绿色服务业发展。推动服务业集聚发展，力争新增省级服务业集聚区3个。加快“健康宣城、养生福地”建设，大力发展健康养老养生、健身休闲产业。推进“电商宣城”建设，巩固提升农村电商发展水平。引导传统商贸实体企业转型升级，开展消费促进活动。举办宣城2018皖东南名品展销会。大力发展第三方物流，培育或引进A级以上现代物流企业4家。稳妥发展商业保理、互联网金融、现代保险业等新兴金融业态。

（八）提升经济外向水平，打造内陆开放新高地。积极融入“一带一路”等国家战略，全面等高对接沪苏浙，着力形成双向互动、内外联动的全面开放格局。

大力推进对接合作。围绕要素承接、产业发展、制度创新、基础设施共建、公共服务共享、生态环境共治等领域，推进与长三角城市群的深度合作和融合发展。积极承接沪苏浙地区产业转移，加快推进宣宁产业园建设及白茅岭、军天湖沪宣“飞地”合作，争取苏皖合作示范区建设规划获批实施。推进与粤闽港澳台地区合作发展。

持续加强招商引资。开展“招商引资质量年”活动，进一步完善和落实招商引资政策，强化对已落户企业服务，确保开工、竣工、投产项目数和形成固定资产投资额均增长10%以上，实际利用外资增长6.5%左右；力争在国内外500强企业、行业50强企业招引上实现新的突破。强化统筹调度，整合驻外招商力量，建立市本级与县市区、与园区重大招商项目共引共建共享的联动机制。坚持产业招商，运用“产业+基金+政策+平台”等招商新模式，强化以商招商、产业链招商、市场化招商、精准招商。

促进外贸优化升级。加快内陆二类口岸、宣城现代服务业产业园区综合保税区申报工作。积极推广运用国际贸易“单一窗口”。规范运营保税监管场所，积极落实铁海联运等多式联运模式，支持保税物流做大做强，优化对外贸易结构。加强外贸主体培育，新增进出口实绩企业90家。做大卫浴、汽车零部件、羽绒、农产品等出口基地，支持企业开展国际体系认证，加强出口名牌创建。推进宁国汽车零部件、郎溪食品农产品等出口产品质量安全示范区创建。鼓励企业“走出去”开展国际合作。

（九）加强保障和改善民生，创造宣城人民更加美好的生活。宣城伟大的发展成就由宣城人民创造，应该由宣城人民共享。政府全部工作应以造福人民为最大政绩，尽力而为、量力而行，持续增强全市人民获得感、幸福感、安全感。

加强就业和社会保障。千方百计扩大就业，城镇新增就业人口4万人。深入推进全民参保登记，实现法定人群应保尽保。提高城乡低保标准，完善社会救助制度。深化养老服务体系建设，强化居家养老和社区养老功能，深入开展养老院服务质量建设专项行动。扩大城区住房保障范围，向环卫、公交等行业困难群体倾斜，完成9353户棚户区改造和5615户农村危房改造任务。落实加快残疾人小康进程规划纲要，完善市残疾人康复中心功能。切实维护妇女、儿童权益，加强对农村留守儿童和妇女、老年人的关爱服务。发展社会福利和公益慈善事业。

优先发展教育事业。编制新一轮市区教育建设与发展规划。优化市区教育布局和体制机制。加快实施第三期学前教育行动计划，探索推进普惠制幼儿园建设，解决入园难问题。推进义务教育优质均衡发展。开工建设北师大宣城学校和市第五中学，建成市体校。实施高中教育普及攻坚计划，继续推进宣城中学打造皖南名校。提升特殊教育水平。加强合工大宣城校区基础建设，支持与高水平大学相适应的一流学科建设。加快宣城职业技术学院、皖南医药卫生学校、安徽工业技师学院建设与发展。推进中等职业教育布局结构调整，实施职业教育质量提升工程，促进校企合作办学和中高职衔接培养。完善中小学教师“县管校聘”机制，全面推进教师“无校籍”管理改革，加大义务教育学校校长“职级制”改革。加强教育师德培养。鼓励社会力量兴办教育，促进民办教育健康发展。

推进健康宣城建设。实施“健康宣城2030”规划纲要，启动省级健康城市创建试点工作，促进医药卫生事业发展。加快推进市人民医院、省立医院皖南分院（市妇幼保健院）、市中医院、北京协和医院宣城皖东南心血管医院、中心血站血液检测楼、市公共卫生检验检测中心等项目建设，实施乡镇卫生院分类管理，完善医疗卫生服务体系，努力让群众在家门口享受到优质医疗服务。加强重大疾病防控。加快计划生育转型发展，拓展优质服务。创建食品药品安全城市，强化食品药品市场监管。广泛开展全民健身活动，推进体育强市建设。以更强实力参加省第十四届运动会。

繁荣发展文化事业。践行社会主义核心价值观，提升全国文明城市创建成果。加快推进市级艺术团体组建和艺术剧院建设，扩大乡镇（社区）综合文化服务中心、农民文化乐园覆盖面，力争实现农家书屋数字化全覆盖。加强文化场所管理。推进国家历史文化名城创建，加强文物保护和古建筑古村落保护利用。创成中国书法城，申创中国诗歌城。继续推进市国家综合档案馆建设。支持郎溪与溧阳共同打造傩文化保护基地。推进传统媒体与新兴媒体深度融合发展。

加强和创新社会治理。坚持平安为基，继续推进全国社会治安综合治理优秀市创建，打造共建共治共享的社会治理格局。深入实施“雪亮工程”，建设立体化、信息化、智能化社会治安防控体系。持续开展守护平安系列行动，深入推进“一村一警一辅”包村联系工作，依法打击各类违法犯罪活动。深化基层综治中心“国标化”建设，完善村社区网格员“六全”工作法。深化司法行政改革，加大全民普法力度，建设社会主义法治文化，推进市禁毒基地、道路交通安全教育基地和法治宣传教育基地建设。认真做好法律援助、社区矫正和安置帮教工作。强化信访源头治理，开通全市视频接访系统，坚持领导开门接访、带案下访、主动约访，全力化解社会矛盾。推进安全生产领域改革发展，深入开展隐患排查和专项整治，推进安全文化建设，加大安全知识宣传教育力度，有效防范和遏制较大以上事故。加强气象、地震、消防等工作，提高综合防灾减灾救灾和应急处置能力。推动民族团结进步，依法加强宗教事务管理。支持群团组织改革发展。做好村民委员会、城市社区换届选举工作。发挥社区自治作用，提高社区自治水平。扎实开展第四次全国经济普查，完成第二次全国地名普查。加强新形势下全民国防教育、国防动员、人民防空和双拥优抚安置工作，争创新一轮全国双拥模范城，巩固和促进军政军民团结。

在高质量完成省定民生工程的基础上，市政府还将办好10项民生实事：①老旧小区电气整治工作；②新改扩建幼儿园12所；③实施新生儿疾病筛查；④推进“三车”运输秩序整治和文明交通建设；⑤规范和提升住宅小区物业管理水平；⑥市区老旧小区供水“一户一表”改造；⑦市区老旧小区天然气改造；⑧对城区“三无”对象中特困供养人员发放居家养老护理补贴；⑨市区住宅小区把式停车棚建设；⑩市区保障性蔬菜基地能级提升工程。

三、全面加强政府自身建设

事业关键在人，发展必须实干。站在历史新方位，开启发展新征程，迫切需要我们全面从严加强政府自身建设，提高履职尽责能力和水平，充分展现新时代政府工作新气象和新作为。

一要旗帜鲜明讲政治。把学习宣传贯彻党的十九大精神作为首要政治任务，在学懂弄通做实上下功夫，自觉用习近平新时代中国特色社会主义思想武装头脑、指导实践、推动工作。扎实开展“不忘初心、牢记使命”主题教育，强化“四个意识”，严守纪律规矩，坚决维护习近平同志核心地位，坚决维护党中央权威和集中统一领导。全面加强学习和实践，增强执政能力，培养专业能力，坚定不移推动党的十九大精神在宣城落地生根，坚定不移地贯彻落实省委、省政府和市委的决策部署。

二要履职尽责重法治。推进依法行政，加快建设法治政府。坚持科学民主依法决策，实现重大行政决策合法性审查和公平竞争审查全覆盖。加强政府立法能力和机制建设，提高政府立法水平。推进行政执法体制改革，严格规范公正文明执法。运用法治思维和法治方式推动工作。自觉接受党内监督、市人大及其常委会法律监督和工作监督、市政协民主监督，主动接受司法监督、群众监督和舆论监督。加大政务公开力度，推进行政行为阳光透明。加强政务诚信建设，进一步增强政府公信力。

三要持之以恒提效能。提升“互联网+政务服务”水平，推进政务服务事项网上办理，做到“应上尽上、全程在线”。建立网上审批负面清单制度，整合审批窗口，完善窗口受理无否决权、容缺受理机制，打造“马上就办”服务品牌，实现“一张网一个门、群众办事不求人”，创优“四最”营商环境。落实经济运行和重点工作调度、双月观摩调度、督查通报、“无会周”外出招商等制度，完善绩效考核评价体系，运用好政务综合管理平台，加强政务督查和目标管理考核，加大对庸政懒政怠政行为追责问责力度，保障工作高效落实。

四要真抓实干勇担当。幸福都是奋斗出来的。加快新时代宣城高质量发展，必须不驰于空想、不骛于虚声，勇于冲破思想观念障碍，勇于摒弃惯性思维，始终保持锐意进取的精神风貌，始终保持奋发有为的工作状态，始终踏踏实实干好工作。大力弘扬“大国工匠”精神和新时代宣城创建精神，切实增强狠抓落实本领，争当实干兴宣的行动英雄，践行一线工作法，往前站、随我上、带头干，身入心至、直面矛盾、担当尽责，崇尚实干、力戒空谈、精准发力，以钉钉子精神和“绣花功夫”落细落小落实，坚决防止和杜绝表态多调门高、行动少落实差的现象发生。进一步健全激励和容错纠错机制，为大胆创新探索者撑腰鼓劲。

五要心存敬畏守底线。全面履行从严治党主体责任，深入推进政府系统党风廉政建设和反腐败斗争。坚持“一岗双责”、以上率下，发挥好“头雁”效应。完善政府权力运行事中事后监管，健全监督制约机制，以公共资金、国有资产资源、机构编制等为重点，全面加强规范管理，扎紧织密制度笼子。自觉接受巡视巡察监督，强化政府内部层级监督和审计监督。以永远在路上的韧劲强化作风建设，严格落实中央八项规定实施细则精神和省市实施细则要求，持之以恒正风肃纪，坚决防止“四风”反弹回潮。全力支持纪检监察机关履行职责，开展扶贫领域腐败和作风建设专项整治，坚决查处侵害群众利益的不正之风和腐败问题。

各位代表，同志们！新时代开启新征程，新时代呼唤新作为。让我们更加紧密地团结在以习近平同志为核心的党中央周围，在中共宣城市委的坚强领导下，不忘初心、牢记使命，勇于担当、砥砺奋进，为决胜全面建成小康社会、加快打造现代化“四个特色之城”、奋力开创新时代建设皖苏浙省际交汇区域中心城市新局面、实现宣城人民对美好生活的向往而不懈奋斗!
